# *In vivo* anti-hyperlipidemic activity of the triterpene from the stem bark of *Protorhus longifolia* (Benrh) Engl

**DOI:** 10.1186/1476-511X-13-131

**Published:** 2014-08-15

**Authors:** Kgothatso E Machaba, Sinazo ZZ Cobongela, Rebamang A Mosa, Lawal A Oladipupo, Trayana G Djarova, Andy R Opoku

**Affiliations:** Department of Biochemistry and Microbiology, University of Zululand, Private Bag X1001, KwaDlangezwa, 3886 Republic of South Africa; Natural Products Research Unit, Lagos State University, Ojo, Lagos, Nigeria

**Keywords:** Hyperlipidemia, High fat diet, Triterpene

## Abstract

**Background:**

Hyperlipidemia, a metabolic disorder of lipids, is a well known risk factor of cardiovascular events and metabolic syndrome. In this study, the *in vivo* lipid-lowering activity of the triterpene (Methyl-3β-hydroxylanosta-9,24-dien-21-oate), isolated from the stem bark of *Protorhus longifolia,* in high fat diet (HFD)-induced hyperlipidemic rats was investigated.

**Methods:**

Structure of the isolated compound was established and confirmed based on spectral (NMR, HRMS, IR) data analysis. Rats were divided into two groups; normal group (fed the normal commercial rats’ chow) and the HFD group. After 21 days of experimental period on their respective diets, the HFD rats were sub-divided into 4 groups of six rats per group. Two of the HFD groups were orally treated with the triterpene (100 and 200 mg/kg body weight) for 15 days. At the end of the experimental periods, the rats were sacrificed and blood samples were collected for biochemical assays.

**Results:**

The results show that there were significant increases in total serum cholesterol (TC, 15.72 mmol/L) and low-density lipoprotein cholesterol (LDL-c, 7.41 mmol/L) with a reduction in high-density lipoprotein cholesterol (HDL-c, 14.75 mmol/L) in HFD-induced hyperlipidemic rats after 21 days. Oral administration of the triterpene (100 mg/kg.bw and 200 mg/kg.bw) for a period of 15 days resulted in significant lowering of the levels of TC (7.51 mmol/L) and LDL-c (4.46 mmol/L) with an increase in HDL-c (47.3 mmol/L) in HFD-induced hyperlipidemic rats. Significant decrease in atherogenic index and coronary risk index by the triterpene was observed in HFD-induced hyperlipidemic rats.

**Conclusions:**

The triterpene could effectively reduce or control the amount of serum cholesterol and LDL. It is apparent that the compound could contribute to new formulation with significant hypolipidemic effects.

**Electronic supplementary material:**

The online version of this article (doi:10.1186/1476-511X-13-131) contains supplementary material, which is available to authorized users.

## Background

Hyperlipidemia, a disorder of lipid metabolism characterized by elevated levels of lipids circulating in the blood, has now become a global concern. It is considered as one of the five leading causes of death in the world [1]. Its prevalence is greatly influenced by adaptation of sedentary lifestyle and an increase in consumption of a high-fat diet [2]. Hyperlipidemia is strongly linked to the development of cardiovascular events and metabolic syndrome diseases [3]. Thus, regulation of blood lipid levels is vital in the prevention and treatment of hyperlipidemia and its related diseases.

Currently, a number of anti-hyperlipidemic agents have been introduced for the treatment of hyperlipidemia. One of the most widely used anti-hyperlipidemic agents at present is lovastatin, which reportedly slows down the body’s ability to make cholesterol by targeting hepatocytes and inhibiting HMG-CoA reductase; the enzyme that converts HMG-CoA into mevalonic acid, a cholesterol precursor [4]. However, this drug is associated with undesirable side effects (such as myositis and rhabdomyolysis, elevated CK levels, muscle weakness, and muscle cramps) in humans [5, 6]. Such adverse effects of the current anti-hyperlipidemic drugs stimulate the search for alternative medicine with improved efficacy and safety profile.

Medicinal plants have always been rich sources of biologically active compounds vital to human health. Thus a search for new lead molecules with anti-hyperlipidemic properties from plants could be a useful strategy. Anti-hyperlipidemic activity of a number of medicinal plants and/or plant-derived bioactive compounds has been reported. Such studies include the hypolipidemic activity of *Pandanus tectorius* fruit extract [7], *Coriolus versicolor*[6] and 2, 4, 6-Trihydroxyacetophenone isolated from *Myrciamultiflora*[8]*.*

Stem bark of *Protorhus longifolia* (Benrh.) Engl. (Anacardiaceae), a tree indigenous to Southern Africa, is one of the plants commonly used by Zulu traditional healers in the management of blood-clotting related diseases. Antimicrobial activity of the leaf extracts of the plant has been reported [9]. Cytotoxic and anti-platelet aggregation activity of the crude extracts and two lanosteryl triterpenes from the stem bark of the plant have recently been reported [10, 11]. A growing body of evidence support plant-derived triterpenes as new targets for drug development due to their diverse potential pharmacological activities. The *in vitro* anti-hyperlipidemic activity of methyl-3β-hydroxylanosta-9,24-dien-21-oate isolated from *Protorhus longifolia* has been observed in our laboratory (unpublished data). The present study reports on the *in vivo* hypolipidemic activity of the triterpene from *Protorhus longifolia* in a high fat diet (HFD)-induced hyperlipidemia in rats.

## Results

**Figure 1:**
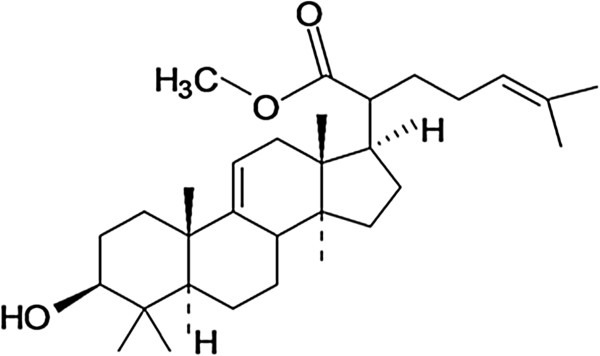
**Chemical structure of methyl-3β-hydroxylanosta-9,24-dien-21-oate (KE1).**

The structure of the isolated compound (Figure 1) was established and confirmed using ^1^H and ^13^C-NMR. The isolated compound (Methyl**-**3β-hydroxylanosta-9,24-dien-21-oate, **KE1**) was obtained as white crystals, > 95% pure, mp 204-205°C, IR (KBr) *v*_max_ = 3469, 1683 cm^−1^. See Table 1 for ^1^H and ^13^C NMR data. The data suggested the molecular formula C_31_H_50_O_3_, calculated 470.736. Spectra of the compound are presented in Additional file 1.

**Table 1:** ^**13**^ **C-NMR data and significant** ^**1**^ **H-NMR data of the compound**

Position	δ _C_ (ppm)	Type	δ _H_ (ppm)
1	30.4	CH_2_	
2	23.9	CH_2_	
3	74.3	CH	4.25 (1H, s, OH)
4	37.4	C	
5	44.4	CH	
6	17.3	CH_2_	
7	25.8	CH_2_	
8	49.6	CH	
9	145.9	C	
10	34.7	C	
11	118.3	CH	5.11 (1H, t)
12	28.6	CH_2_	
13	43.3	C	
14	51.0	C	
15	31.3	CH_2_	
16	27.0	CH_2_	
17	47.7	CH	
18	13.3	CH_3_	
19	21.7	CH_3_	
20	48.4	CH	
21	177.3	C	
22	34.7	CH_2_	
23	25.5	CH_2_	
24	125.6	CH	5.21 (1H, t)
25	136.4	C	
26	17.4	CH_3_	1.61 (3H, s)
27	25.8	CH_3_	1.65 (3H, s)
28	21.9	CH_3_	1.21 (3H, s)
29	27.4	CH_3_	0.90 (3H, s)
30	22.1	CH_3_	1.16 (3H,s)
-OCH_3_	59.8		3.85 (3H, s)

^13^C-NMR- carbon-13 NMR, 1H-NMR- Hydrogen-1(proton) NMR.

The effects of the normal diet (ND) and HFD fortified with different concentrations of KE1 or lovastatin on the body weight, food conversion, food efficiency ratio, liver weights and adiposity level in HFD fed rats over the period of study are shown in Table 2. The control group of rats subjected to ND for 21 and 36 days showed significant increase (p < 0.05) in the body weight by 12.12% and 41.93%, respectively. It is apparent that the rats were gaining weight over the 36 days of study. The control group of rats subjected to HFD for 36 days showed an increase in body weight (13.66%), food conversion and liver weight but a reduction in food efficiency ratio and adiposity level compared to control group of rats subjected to ND for 21 days. Like lovastatin, a standard hypolipidemic drug, treatment with the triterpene at 100 mg/kg.bw, according to HFD (after 36 days), indicated a potential reduction in percentage body weight, food efficiency ratio, liver weights, adiposity level and an increase in food conversion in rats.

**Table 2:** **Effect of the triterpene on percentage weight change, food conversion, food efficiency ratio, liver weights and adiposity level in HFD rats**

Group	Weight change (%)	Food Conversion	Food Efficiency Ratio	Liver (g)	Adiposity level × 10 ^04^
**ND** (after 21 days)	12.12 ± 0.44^***^	0.82 ± 0.07	1.23 ± 0.14^***^	3.37 ± 0.17	1.17 ± 0.03
(after 36 days)	41.93 ± 6.29	0.21 ± 0.02	4.91 ± 0.47	2.97 ± 0.03	1.17 ± 0.04
**HFD** (after 21 days)	6.18 ± 1.09	1.30 ± 0.35	0.96 ± 0.25	4.67 ± 0.06	1.02 ± 0.01
(after 36 days)	13.66 ± 1.77^***^	0.62 ± 0.05	1.53 ± 0.13^***^	5.21 ± 0.29^***^	1.06 ± 0.01^*^
**HFD/KE1** (100 mg/kg)	6.01 ± 1.57	0.75 ± 0.14	1.12 ± 0.40	4.68 ± 0.21	1.04 ± 0.01
**HFD/KE1** (200 mg/kg)	10.04 ± 2.72	0.19 ± 0.02	4.43 ± 1.03***	5.73 ± 0.18	1.08 ± 0.01
**HFD/lovastatin** (10 mg/kg)	3.74 ± 1.20**	2.17 ± 0.59**	0.69 ± 0.14	4.75 ± 0.14	1.03 ± 0.01

All values are expressed as mean ± SEM, (n = 4); *p < 0.05, **p < 0.01, ***p < 0.001 compared to the HFD 36 days.

*p < 0.05, **p < 0.01, ***p < 0.001 compared to the ND 36 days.

Table 3 presents the results on the effects of the HFD and HFD fortified with different concentrations of KE1 and lovastatin on total cholesterol and triacylglyceride levels. The hyperlipidemia group of rats subjected to HFD for 21 and 36 days indicated an increase (p < 0.001) in total cholesterol and LDL with increases in HDL levels. A statistically significant reduction (p < 0.001) in total cholesterol and LDL accompanied by a significant increase in HDL was observed in the animals fed the HFD fortified with the triterpene at 100 and 200 mg/kg.bw according to HFD (after 36 days). There were no statistically significant changes observed in triacylglyceride and VLDL levels compared to normal group of rats fed ND for 36 days.

**Figure 2:**
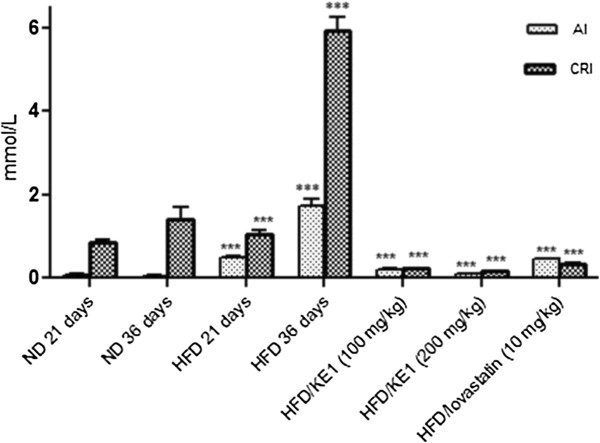
**Effect of the triterpene on atherogenic index (AI) and coronary risk index (CRI) in HFD-induced hyperlipidemia in rats.** All values are expressed as mean ± SEM, (n = 4); ***p < 0.001 compared to the HFD 36 days; ***p < 0.001 compared to the ND 36 days.

The effect of the triterpene on atherogenic index and coronary risk index in hyperlipidemic rats was also evaluated and the results are given in Figure 2. The triterpene at different concentrations (100 and 200 mg/kg.bw) exhibited a significant reduction in atherogenic index and coronary risk index in HFD-induced hyperlipidemic animals for 36 days. Similar results were also observed in the rats fed HFD fortified with lovastatin.

**Table 3:** **Effect of the triterpene on serum lipids and lipoproteins levels (mmol/L) in HFD-induced hyperlipidemia in rats**

Group	Total cholesterol	Triglyceride	VLDL-c	LDL-c	HDL-c
**ND** (after 21 days)	1.38 ± 0.12	0.68 ± 0.11	0.14 ± 0.04	0.11 ± 0.04	1.14 ± 0.90
(after 36 days)	1.83 ± 0.24	0.79 ± 0.06	0.26 ± 0.18	0.10 ± 0.02	1.86 ± 0.07
**HFD** (after 21 days)	15.72 ± 1.10	1.29 ± 0.54	0.28 ± 0.22	7.41 ± 0.56**	14.75 ± 0.19***
(after 36 days)	49.52 ± 4.83^***^	1.41 ± 0.35	0.28 ± 0.14	10.72 ± 0.94^***^	6.17 ± 0.16^***^
**HFD/KE1** (100 mg/kg)	34.24 ± 1.50***	1.93 ± 0.15	0.39 ± 0.05	5.85 ± 0.81***	30.39 ± 0.92***
**HFD/KE1** (200 mg/kg)	7.51 ± 0.97***	0.46 ± 0.05	0.09 ± 0.01	4.46 ± 0.82***	47.30 ± 0.9***
**HFD/lovastatin** (10 mg/kg)	38.66 ± 3.25*	0.77 ± 0.13	0.15 ± 0.03	12.97 ± 0.59	29.13 ± 0.68***

All values are expressed as mean ± SEM, (n = 4); *p < 0.05, **p < 0.01 compared to the HFD 36 days; *p < 0.05, **p < 0.01, ***p < 0.001 compared to the ND 36 days.

**Figure 3:**
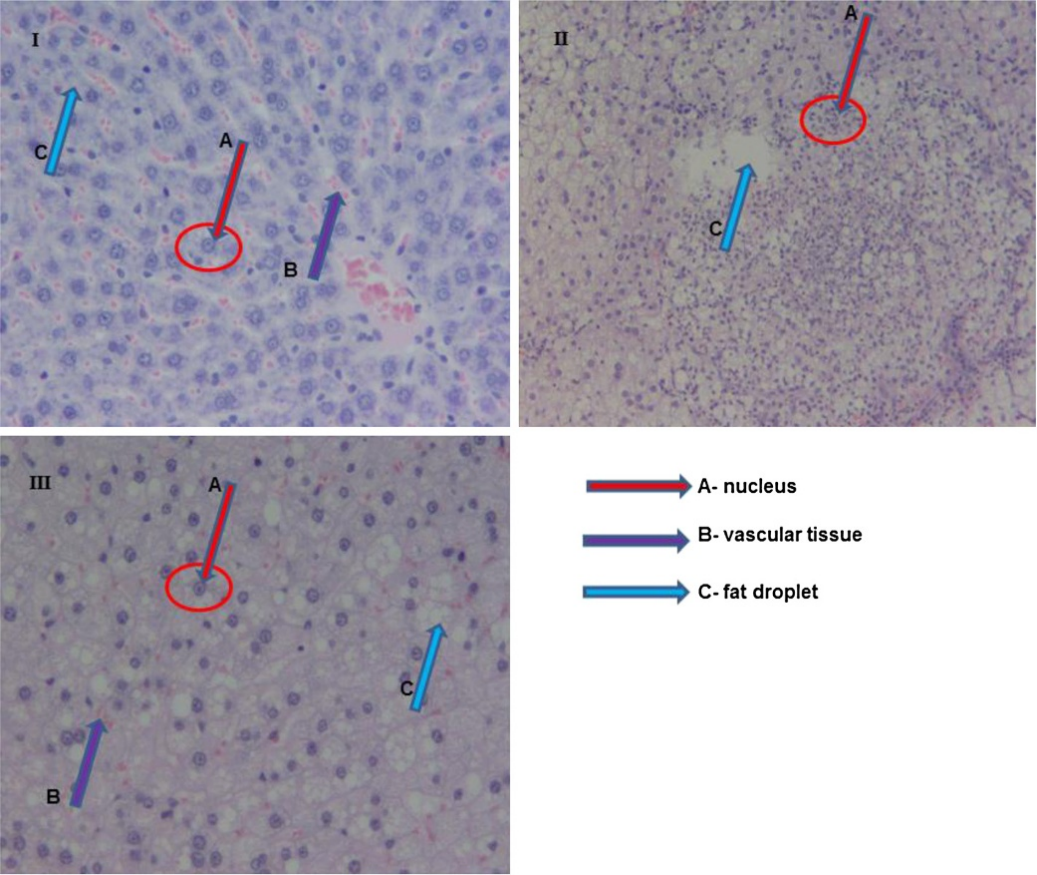
**Histopathological changes (200x magnification) in liver of high fat diet induced hyperlipidemia after 15 days treatment with the triterpene. I**- liver tissue of the rat fed a normal diet; **II**- liver tissue of the rat fed a HFD; **III**- liver tissue of the triterpene (200 mg/kg.bw) treated and HFD fed rat.

The results for liver enzymes (ALP, ALT and AST) levels along with histopathological changes of the rat liver following treatment with HFD fortified with the triterpene are presented in Table 4 and Figure 3, respectively. Reduced levels of the liver enzymes were observed in rats treated with HFD fortified with the triterpene reaching levels similar to those in the ND group. Liver tissue from the rat treated with the triterpene depicted a lower accumulation of lipid droplets as compared to the liver of the rat fed HFD only in which numerous fat droplets and severe vascular changes with displaced nuclei were evident.

**Table 4:** **Effect of the triterpene on liver enzymes on HFD-induced hyperlipidemia in rats**

Group	ALP (U/L)	ALT (U/L)	AST (U/L)
**ND** (after 21 days)	73.00 ± 24.85	45.90 ± 13.45	226.00 ± 95.57
(after 36 days)	46.10 ± 11.94	51.90 ± 10.32	289.20 ± 68.95
**HFD** (after 21 days)	179.90 ± 34.38	78.20 ± 30.09	339.30 ± 138.06
(after 36 days)	249.00 ± 60.40^**^	83.80 ± 18.67	298.80 ± 55.34
**HFD/KEM** (100 mg/kg)	181.10 ± 25.53	42.30 ± 6.90	161.90 ± 52.22
**HFD/KEM** (200 mg/kg)	363.30 ± 53.23	48.80 ± 8.28	205.50 ± 37.12
**HFD/lovastatin** (10 mg/kg)	108.80 ± 35.03	44.11 ± 9.82	188.80 ± 52.76

All values are expressed as mean ± SEM, (n = 4); **p < 0.01, compared to the ND 36 days.

## Discussion

The structure of the isolated compound (Figure 1) was established and confirmed on the basis of ^1^H and ^13^C NMR data analysis. The ^1^H-NMR of the compound followed the same triterpenoid pattern observed in 3β-hydroxylanosta-9,24-dien-21-oic acid [11], with a large clusters of signals of CH_3_, CH_2_ and CH between δ_H_ 2.5 and 0.8. The ^13^C-NMR of this compound also resembled that of 3β-hydroxylanosta-9,24-dien-21-oic acid, with five quaternary carbon atoms, and the presence of four olefinic carbon atoms between 145–118 ppm confirming the lanosteryl skeletal structure. The presence of an ester carbon atom at δ_C_ 177.3 instead of a carboxylic carbon at δ_C_ 181.5 assisted in suggesting the methyl ester of 3β-hydroxylanosta-9,24-dien-21-oic acid. Detailed assignment of the ^13^C-NMR and significant ^1^H-NMR of the compound are presented in Table 1. The absorption bands for carbonyl (1683 cm^−1^), and hydroxyl (3469 cm^−1^) functional groups observed on IR spectrum also further assisted in confirming the NMR structure.

Consumption of a high-lipid diet may create diverse patterns of hyperlipidemia. Abnormalities of lipid metabolism are associated with cardiac diseases, obesity and their associated disorders [12]. Thus regulation of dyslipidemia is crucial for the prevention and treatment of cardiovascular events [13, 14].

In this study, the hypolipidemic activity of the triterpene from stem bark of *P. longifolia* was evaluated in the HFD-induced hyperlipidemia in rats. The results obtained from this study demonstrate that the triterpene has anti-hyperlipidemic properties. The ability of the HFD supplemented with the triterpene to significantly (p < 0.001) lower serum TC and LDL-c levels while increasing HDL-c levels (Table 3) suggested the hypolipidemic activity of the compound. These results are consistent with other literature reports on the hypolipidemic activity of plant-derived compounds. A triterpenoid mixture (α, β-amyrin) from *Protium heptaphyllum*[15] and lanostane triterpenoids from *Prosthechea michuacana*[16] have been reported to exert their hypolipidemic effects through reduction of serum cholesterol and triglyceride while increasing the levels of HDL-c. Consumption of plant sterol and their esters has also been reported to not only lower intestinal cholesterol absorption but decreased blood levels of the atherogenic LDL-c as well [17, 18]. Since HDL possesses many features that contribute to the protection from atherosclerosis and related incidences [19], hypolipidemic agents with the ability to also increase serum levels of HDL-c are of great target.

The lower AI and CRI (Table 3) observed in this study in HFD-induced hyperlipidemic rats following the 15 days treatment with the triterpene suggested the cardiovascular protective potential of the compound. The formation of plaque in arteries, atherosclerosis, as a result of high cholesterol and LDL-c in the blood can lead to serious problems such as stroke, heart attack or even death [20]. Significant reduction in AI and CRI were also reported by Chaudhari *et al.*[12] using embelin extracted from *Embelia ribes* and this is considered beneficial in patients with atherosclerosis and obesity. The triterpenoid mixture from *Protium heptaphyllum* has also been reported to significantly reduce the AI in HFD-induced hyperlipidemia in rats [15]. It is apparent that the compound exerts its therapeutic effect through reduction of atherogenic cholesterol and triglyceride.

Prolonged consumption of high-fat diet increases synthesis of TG and inhibit β-oxidation of fatty acids which consequently leads to the accumulation of excess TG in the liver [12, 21]. The accumulation of the TG causes an increase in liver weight and adipose tissues [12, 22]. Interestingly, the triterpene from *P. longifolia* did not only reduce body weight, but liver weight and adiposity level (Table 2) in rats. The reduction in the liver enzymes activity (Table 4) and accumulation of fat droplets (Figure 3) in the liver of HFD-induced hyperlipidemic rats is indicative of the hepatoprotective effect of the triterpene with low cytotoxicity. Elevated liver enzymes may indicate inflammation or damage to cells in the liver [23]. Thus liver enzymes are the proper indicator of normal function of the liver. A low cytotoxicity of the triterpenes from *P. longifolia* on hepatic and kidney cells has been reported [11].

## Conclusion

The results obtained in this study show that the triterpene (methyl-3β-hydroxylanosta-9,24-dien-21-oate) isolated from *Protorhus longifolia* has a significant hypolipidemic activity in high fat diet fed rats. This is evidenced by the reduction of serum TC and LDL-c, with an increased HDL-c concentration in the HFD fed treated groups. Significant reduction in AI and CRI by the triterpene was observed and it can thus be considered beneficial in reducing the risk of atherosclerosis. The compound could effectively control the amount of serum lipids and liver enzymes with lower toxicity. It is apparent that the compound could contribute to new formulation with significant hypolipidemic effects.

## Methods

### Plant material

Fresh stem barks of *Protorhus longifolia* (Benrh.) Engl. were collected in March 2012 from Hlabisa, Kwa-Zulu Natal, South Africa. The plant (voucher specimen number RA01UZ) was authenticated by Dr. N.R. Ntuli, Department of Botany, University of Zululand. The plant material was thoroughly washed with tap water and then air dried. The air dried plant material was ground into powder (2 mm mesh) and stored in sterile brown bottles until use.

### Extraction and isolation

The method of extraction and isolation of the triterpenes from the stem bark of *P. longifolia* has been previously described [11]. Briefly, the powdered plant material was first defatted with *n*-hexane and then extracted (1:5 w/v) with chloroform. The compounds were isolated from the chloroform extract (13 g) using silica gel column chromatography (24 × 700 mm; Silica gel 60; 0.063 - 0.2 mm; 70–230 mesh ASTM, Merck, Darmstadt, Germany). The column was eluted stepwise with a mixture of *n*-hexane and ethyl acetate (9:1–3:7) and 20 ml fractions were serially collected. Thin layer chromatography (TLC) (silica gel 60 TLC aluminium sheets 20 cm × 20 cm, F_254_, Darmstadt, Germany) was used to analyse the fractions. The combined fraction 14 was further purified in ethyl acetate to afford the compound (**KE1**, 1.15 g). Melting point of the compound was determined using Stuart SMP 11 melting point apparatus (Shalom Instruments supplies, Durban, South Africa). Spectroscopic data analysis, NMR (^1^H-^1^H, ^13^C-^13^C, in DMSO, Bruker 600 MHz), HRMS (in DCM, Waters Synapt G2) and infrared (IR) (Perkin-Elmer 100 FTIR) techniques were used to establish and confirm structure. Chemical shifts were expressed in δ (ppm).

### Animals

This study was approved by University of Zululand Research Animal Ethics Committee (UZREC 171110–030 Dept. 2013/23). Sprague–Dawley rats (180-220 g) were collected from animal house in the Department of Biochemistry, University of Zululand, South Africa. Experimental procedures were conducted following the guideline for care and supervision of experiments on animals. The animals were housed in standard cages and maintained at room temperature with 12:12-h light: dark cycle. All rats had free access to drinking water and standard rat feed in the experimental environment, for 1 week, before the experiment was conducted. Once the animals had adapted to the environment, forty-two (42) rats were divided into two groups; normal group (ND) consisting of 12 rats and the high fat diet (HFD) group consisting of 30 rats. After 21 days on their respective diets, 6 rats per group were sacrificed and the remaining rats of the HFD group were randomly divided into a total of four (4) groups of six rats per group.

### Experimental design

1. Group 1: normal diet and vehicle throughout the study
2. Group 2: high fat diet and vehicle throughout the study
3. Group 3: was subdivided into two groups (A, B) and received high fat diet and compound (100 and 200 mg/kg body weight, respectively), dissolved in 2% Tween 20
4. Group 4: high fat diet and lovastatin (10 mg/kg body weight), dissolved in 2% Tween 20

### Induction of dyslipidemia in rats

The method previously described by Hor *et al.*[6] was followed to evaluate the anti-hyperlipidemic activity of the triterpene. Rats were made hyperlipidemia by feeding a high fat diet [commercial rat chow (97.3%), Sunflower oil (15%), bile salt (0.5%), cholesterol (5%), Thirmecil (0.2%)]. This HFD preparation was pelleted (about 3 g each) and fed daily to the rats for 36 days to induce hyperlipidemia.

### Measurement of body weight and food intake

Body weight and food intake were recorded every other day over the study period of 21 or 36 days. Percentage weight change, food conversion (FC), and food efficiency ratio (FER) were then calculated.


where wt- weight

### Collection of blood samples and Liver for Lipid profile determination

At the end of the experimental periods, the rats were fasted for 8 hours, and then sacrificed by a blow to the head and blood samples were collected by cardiac puncture. The collected blood samples were centrifuged at 3500 rpm for 10 minutes and the serum collected for biochemical studies. The liver was excised, weighed and stored in formalin for histological studies.

Histology studies were carried out at the Vet Diagnostix Laboratories (Pietermaritzburg, SA) by qualified pathologist having no prior knowledge to which group they belonged. The liver tissues were stained with haematoxylin and eosin (H & E). This method allowed for unbiased description of the histological lesions which were present or absent in the samples.

### Biochemical assays

The serum samples were used for the estimation of total cholesterol (TC), total triglyceride (TG), HDL-cholesterol (HDL-c), AST, ALT and Alkaline phosphatase. Analysis was done using the Cobas c 111 analyzer.

LDL-cholesterol (LDL-c) was estimated using Friedwald’s equation [24]

LDL-c = [TC-(HDL-c + (TG/5)]

Other lipids parameters such as VLDL- cholesterol, coronary risk index and atherogenic index (AI) were calculated [12] as follows:

LDL-c = [TC-(HDL-c + (TG/5)]

VLDL-c = [TG – (HDL-c + LDL-C)]

Atherogenic index (AI) = LDL-c/HDL-c (mg/dl)

Coronary risk index (CRI) = TC/HDL-c (mg/dl)


### Statistical analysis

Data was analyzed using one-way analysis of variance (ANOVA) followed my Tukey-Kramer multiple comparison test using GraphPad InStat® version 3. The results are presented as mean ± standard error of the mean (SEM). Values of p < 0.05 were considered significant.

## Electronic supplementary material

Additional file 1: **A1 Spectra of KE1. Figure S1.** IR spectrum of KE1. **Figure S2.** ^1^H-NMR spectrum of KE1. **Figure S3.** ^13^C-NMR spectrum of KE1. (DOC 3 MB)

## Abbreviations

AI: Atherogenic index
ANOVA: One-way analysis of variance
CRI: Coronary risk index
CVD: Cardiovascular diseases
FC: Food conversion
FER: Food efficiency ratio
HDL: High density lipoproteins
HFD: High fat diet
IR: Infrared
LDL: Low density lipoproteins
HRMS: High resolution mass spectrometry
ND: Normal group
NMR: Nuclear magnetic resonance
TG: Triacylglycerol
TLC: Thin layer chromatography
VLDL: Very low density lipoprotein
ALT: Alanine aminotransferase
AST: Aspartate aminotransferase.
