# Supplementary material for: In vivo anti-hyperlipidemic activity of the triterpene from the stem bark of Protorhus longifolia (Benrh) Engl
Source: Lipids Health Dis. 2014 Aug 15;13:131. doi: 10.1186/1476-511X-13-131 (PMC4246574; doi:10.1186/1476-511X-13-131)
Supplement: Supplementary file 1 — Additional file 1: A1 Spectra of KE1. Figure S1. IR spectrum of KE1. Figure S2. 1H-NMR spectrum of KE1. Figure S3. 13C-NMR spectrum of KE1. (DOC 3 MB) [file 12944_2014_1154_MOESM1_ESM.doc]

**Additional file**

**A1. Spectra of KE1**

**Figure S1:** IR spectrum of KE1

**Figure S2:** ^1^H-NMR spectrum of KE1

**Figure S3:** ^13^C-NMR spectrum of KE1
